# A BOSSS platform: using functionalized lipids and click chemistry for new discoveries in lipid research

**DOI:** 10.1016/j.jlr.2021.100025

**Published:** 2021-01-16

**Authors:** David A. Ford

**Affiliations:** Edward A. Doisy Department of Biochemistry and Molecular Biology and Center for Cardiovascular Research, Saint Louis University School of Medicine, St. Louis, MO, USA

The development of new synthetic reporter lipids is critical in our continued pursuit to understand the intricacies of complex lipid physical and biological properties. Reporter functionalized lipids include, but are not limited to, fluorescently labeled lipids, electron paramagnetic probe-labeled lipids, and MRI lipids. Other functionalized lipids include those synthetic lipids (e.g., polyethylene glycolated lipids) used for drug delivery and gene transfection. Although these functionalized lipids are important reagents in the lipid biochemist's toolbox, the investigator must also consider that the employed functionalized lipid may not always necessarily mimic the natural lipid of interest. This limitation can be reduced with improved functional lipids including lipid click chemistry tools, which have evolved over the past 20 years (1) (Fig. 1).

**Fig. 1:**
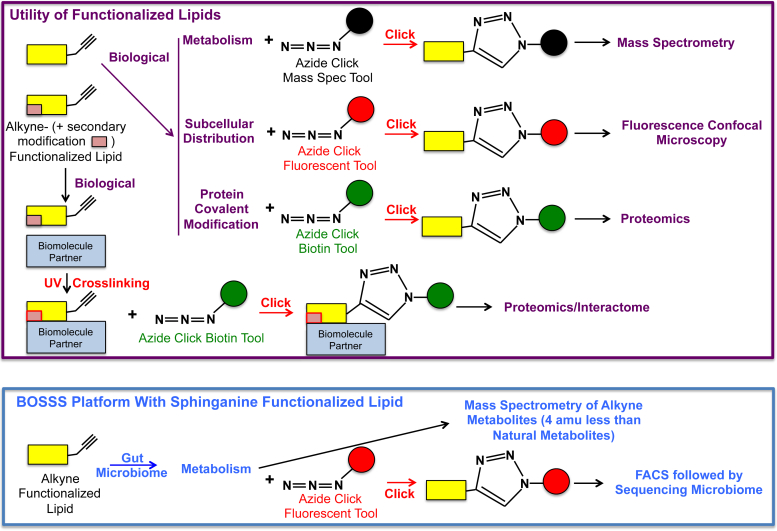
Functionalized lipids with click chemistry and photoactivatable modifications. Functionalized lipids have been used to characterize lipid metabolism, subcellular distribution, and protein covalent modification using click chemistry analogues. Lipid click chemistry analogues may have either alkyne or azide modifications (*yellow rectangle* with alkyne shown), which then can be clicked to click chemistry tools containing either an azide or alkyne, respectively. These tools may contain either fluorescent probes (*red circle*), biotin (*green circle*), or mass spectrometry probes (*black circle*) to enable protein modification, subcellular localization, proteomics, and metabolite identification. Furthermore, click chemistry lipid analogues with secondary photoactivatable modifications (*pink rectangle* within *yellow rectangle*) can be used to covalently bind to interacting biomolecule partners by UV cross-linking. The Elizabeth Johnson laboratory has added a new dimension to the utility of click chemistry lipid analogues described as the BioOrthogonal Sorting, Sequencing and Spectrometry (BOSSS) platform. Alkyne-modified sphinganine provided to the microbiome can be directly used to discriminate between the metabolism of natural sphinganine and alkyne-modified sphinganine because of the loss of four hydrogens. The novelty of the BOSSS platform was through exploiting a fluorescent click tool to fluorescence-activated cell sorting the microbiome followed by sequencing the sorted fluorescently labeled microbes to determine microbe speciation.

Bioorthogonal click chemistry provides lipid researchers a powerful tool to investigate lipid metabolism, lipid subcellular distribution, and lipid interactions with other biomolecules. Using bioorthogonal click chemistry, a biological system can be treated with a biomolecule analogue modified with a reactive azide or alkyne distantly positioned from the functional group of the biomolecule (e.g., in the omega position of a fatty acid), which subsequently can be clicked to a reporter molecule containing a respective alkyne or azide. The ligation of the biomolecule analogue with the reporter is generally catalyzed by the copper-mediated Huisgen cycloaddition reaction (2). Live-cell studies can also be performed using bioconjugations including azides with cyclooctynes, which do not require cell-toxic copper (3). Bioorthogonal click chemistry has been used in several studies reported in the *Journal of Lipid Research* over the past 2 years to characterize intracellular cholesterol trafficking, identify 2-chlorofatty acid subcellular localization to endothelial Weibel-Palade bodies, determine disparate protein modification between 2-chlorofatty aldehyde and 2-bromofatty aldehyde, and screen for compromised outer membrane in *Escherichia coli* mutants (4, 5, 6, 7). Additional studies used a bifunctional ceramide analogue that was both clickable and photoactivatable to identify ceramide binding proteins (8, 9). Bifunctional phosphatidylcholine (clickable and photoactivatable) was also used to identify specific contact points of paraoxonase 1 responsible for its interaction with high density lipoprotein (10).

In this issue of the Journal, bioorthogonal click chemistry was cleverly used by Lee *et al.* (11) to interrogate the metabolism of sphinganine in the gut microbiome using a strategy that advanced the utility of click chemistry by incorporating flow cytometry and rRNA sequencing into their workflow. This study exploited several key features of click chemistry tools through a protocol that the authors describe as BioOrthogonal-Sort-Sequence-Spectrometry (BOSSS). The BOSSS platform enabled the investigators to identify individual microbial species in the mouse gut microbiome that metabolized sphinganine as well as characterize exogenous sphinganine metabolism by the gut microbiome. Mice were gavaged over 5 days with the bioorthogonal click chemistry analogue of sphinganine containing an omega alkyne (SAA). Subsequently, cecal contents were isolated, and contents were clicked to the fluorescent AF647-azide probe. Next, the gut microbiome was sorted by fluorescence-activated cell sorting (FACS), and then FACS purified SAA containing microbes were identified by 16S rRNA gene sequencing. In addition, mass spectrometry was performed to follow the metabolism of the bioorthogonal click chemistry analogue of sphinganine. This latter approach exploited the utility of the SAA click analogue having a mass 4 amu less than natural sphinganine.

Not surprisingly, the major microbes taking up SAA were the sphingolipid-producing gram-negative *Bacteroides* and *Prevotella*. Interestingly, the nonsphingolipid-producing gram-positive *Bifidobacterium* also incorporated SAA. The major SAA metabolites observed in the cecal microbiome were nonhydroxylated and hydroxylated dihydroceramides. These dihydroceramides were predominantly enriched with aliphatic chains of 15–22 carbons in length. It was also speculated that these molecular species were branched-chain aliphatic groups, which are prevalent in *Bacteroides*. Metabolic comparisons were also determined between *Bacteroides* and *Bifidobacterium* isolated cultures using *Bacteroides thetaiotamicron* (*theta*) and *Bifidobacterium longum infantis*, respectively. Similar to their findings in the cecal microbiome, *B. theta* incorporated SAA into long chain nonhydroxylated and hydroxylated dihydroceramides, whereas *B. longum infantis* only incorporated SAA into very short chain dihydroceramide molecular species. Another significant finding was that *B. theta* incorporated SAA into phosphoethanolamine-containing sphingolipids, which were not observed during the analyses of the cecal microbiome.

The disparate metabolic processing of SAA in isolated cultures compared to the complex gut microbiome suggests that there are intricate regulation and metabolic needs of the microbial species in their cohabitating native environment. Key information will be gained by determining the kinetics of SAA uptake and metabolism by different microbes, which may suggest potential symbiotic relationships. Several questions that are not only limited to microbial sphingolipid metabolism but should be considered globally for gut microbial lipid metabolism can be expanded from these studies employing SAA. For example, do *Bifidobacterium* produce SAA metabolites that are released and then used by other microbes including *Bacteroides*? Are released SAA metabolites from either nonsphingolipid-producing or sphingolipid-producing microbes mediators or biomarkers of disease? Is there differential SAA metabolism by cultured microbes as well as the in vivo gut microbiome in the presence and absence of other dietary carbon sources. Furthermore, it will be important to examine the impact of changes in the gut microbiome elicited by diet or therapeutic intervention on SAA uptake and metabolism by the gut microbiota. It is fascinating to consider the complexity of biochemical mediators potentially signaling between microbial species in respect to microbe metabolism and proliferation, as well as host metabolism and physiology. The workflow of BOSSS can be applied to assess many of these questions.

The studies by Lee *et al.* (11) bring the utility of bioorthogonal click chemistry analogues to the forefront. For lipid research focusing on the gut microbiome, this technique could be used to examine disparate metabolism of fatty acids and complex lipids to better understand disparate roles of the diverse microbiome. In particular, our understanding of the microbiome-host relationship compounded by nutrient utilization is a frontier demanding attention. The BOSSS platform is one tool that can be used to advance our understanding. The BOSSS platform can also be used in many complex systems, including whole animal studies to determine specific cells capable of taking up a lipid. For example, the BOSSS strategy could be used to determine lipid uptake in specific cells present in systemic blood, lymph, and airway. In addition, because of the mass difference compared with the natural lipid species, the metabolism of click analogues can be followed in these complex systems. Another possibility would be to ligate the lipid bioorthogonal click analogue to a mass spectrometry-amenable reporter (e.g., a click tool having a quaternary amine enabling metabolite screening by neutral loss scanning 59.1 amu [loss of the trimethylamine]). Furthermore, as used in the SAA studies, within a cell population, subpopulations may be identified that take up the bioorthogonal click analogue using FACS, and these cells can be compared with others within the same cell population that are click analogue deficient using RNA seek and single-cell sequencing. An interesting application would be to apply this strategy to immune cell diversity in systemic blood. Multiomics could also be applied to define specific metabolic programming differences in cell subpopulations. Many creative investigations are on the horizon as complementary clickable tools are developed, which may enable new insights on the role of lipids in cell biology and human physiology.

## Conflict of interest

The author declares no conflicts of interest with the contents of this article.
